# Small Oligonucleotides Detection in Three-Dimensional Polymer Network of DNA-PEG Hydrogels

**DOI:** 10.3390/gels7030090

**Published:** 2021-07-12

**Authors:** Alessia Mazzarotta, Tania Mariastella Caputo, Luca Raiola, Edmondo Battista, Paolo Antonio Netti, Filippo Causa

**Affiliations:** 1 Center for Advanced Biomaterials for Healthcare@CRIB, Istituto Italiano di Tecnologia (IIT), Largo Barsanti e Matteucci 53, 80125 Naples, Italy; alessia.mazzarotta@gmail.com (A.M.); taniacaputo87@gmail.com (T.M.C.); luca.raiola@gmail.com (L.R.); nettipa@unina.it (P.A.N.); 2 Interdisciplinary Research Centre on Biomaterials (CRIB), Università degli Studi di Napoli “Federico II”, Piazzale Tecchio 80, 80125 Naples, Italy; causa@unina.it; 3 Dipartimento di Ingegneria Chimica del Materiali e della Produzione Industriale (DICMAPI), University “Federico II”, Piazzale Tecchio 80, 80125 Naples, Italy

**Keywords:** PEGDA hydrogels, 3D recognition, diffusion, strand displacement assay

## Abstract

The control of the three-dimensional (3D) polymer network structure is important for permselective materials when specific biomolecule detection is needed. Here we investigate conditions to obtain a tailored hydrogel network that combines both molecular filtering and molecular capture capabilities for biosensing applications. Along this line, short oligonucleotide detection in a displacement assay is set within PEGDA hydrogels synthetized by UV radical photopolymerization. To provide insights on the molecular filter capability, diffusion studies of several probes (sulforhodamine G and dextrans) with different hydrodynamic radii were carried out using NMR technique. Moreover, fluorometric analyses of hybridization of DNA oligonucleotides inside PEGDA hydrogels shed light on the mechanisms of recognition in 3D, highlighting that mesh size and crowding effect greatly impact the hybridization mechanism on a polymer network. Finally, we found the best probe density and diffusion transport conditions to allow the specific oligonucleotide capture and detection inside PEGDA hydrogels for oligonucleotide detection and the filtering out of higher molecular weight molecules.

## 1. Introduction

Hydrogels represent a class of polymers of great interest in many fields, including industrial, biological and biomedical applications [1,2,3,4,5]. Such polymers have a network which, due to their hydrophilic nature, may absorb large amounts of water [6,7,8,9] and physiological fluids without dissolving [6,10,11,12]. The hydrogel network is crosslinked by chemical bond or physical entanglements, which give them the ability to swell or shrink and, in general, alter the overall structure [13,14,15,16,17]. The knowledge and the control at molecular level of a hydrogel structure is then a crucial issue in the network customization to permit the diffusion of a specific target, based on the size and the chemical content. In fact, hydrogels can work as molecular filters representing a physical barrier for large molecules. At the same time, due to the high content of water, in most cases, hydrogels result in antifouling as they are able to repel non-specific binding of proteins from serum. Furthermore, a specific binding can be achieved by adding, during polymerization or in a post modification step, a molecular cue in the bulk or on the surface.

Although hydrogels have been studied for at least three decades, the possibility to exploit the three-dimensional polymeric network as a permselective material for the detection of a specific biomolecular target is still underexplored. In particular, the possibility to exclude interfering biomolecules from the network could pose the basis for a permselective capture. The three-dimensional structure of hydrogels is best described by three parameters: the polymer volume fraction in the swollen state, v_2,s_, the molecular weight between crosslinks, ¯;M_c_, and the mesh size ξ. The study of these parameters and the suitable modulation needed to obtain an engineered material, results in a fundamental understanding of the network structure that allows us to predict the final properties of our materials. Polymer chain fluctuation is an important factor governing solute movement within the hydrogel. In general, the diffusivity of a solute through a crosslinked hydrogel decreases as crosslinking density increases, as the size of the solute increases and as the volume fraction of water within the gel decreases [18]. Because of wide application, in the last years, several methods have been developed to study bulk hydrogel properties, both theoretically and experimentally [12,13,14,15,16,17,18,19,20,21,22,23,24,25,26,27,28,29].

Information can be gained by studying the diffusion of probe molecules through hydrogel networks [30,31,32,33,34,35,36,37]. In the absence of any specific interactions between the probe and the network, the diffusivity of the probe reflects the network structure [29,30,31]. Common techniques used to study probe diffusion include source–sink techniques [32], fluorescence recovery after photobleaching (FRAP) [34,37], FCS [38,39,40] and pulsed field gradient NMR (PFG-NMR) [32,35,41,42,43,44,45]. The key for PFG-NMR analysis is the fact that NMR data render information on the chemical nature, as well as on the molecular mobility, of an observed component. This technique has the potential to characterize water binding and mobility in a directly quantifiable manner, and has been commonly used in studies of the water–polymer interaction [46].

Here we present DNA functionalized PEG crosslinked hydrogels and the investigation of diffusivity of different molecules inside the bulk polymer. The optimizations of crosslinking parameters were carried out with the aim to understand the properties that primarily affect the detection performance of small molecules, such as short oligonucleotides inside the polymer network. Indeed, the high concentration of a wide range of biomolecules could represent an obstacle for recognition, therefore the possibility to use hydrogels as a molecular filter is important to reduce crowding effects within the hydrogels. Therefore, the modulation of both the network structure and capture element density allow to obtain a three-dimensional material for selective target detection. We focus on the synthesis and characterization of PEGDA hydrogels, combining swelling studies with both NMR and fluorometric analysis for diffusion studies. Moreover, the fluorometric analyses of the DNA captured within the hydrogels is investigated in detail to understand the way to better present the immobilized DNA strand for the capture of its complementary counterpart. In such a way, we were able to optimize the network structure to combine both molecular filter and capture element capabilities, to lay the foundation for an engineered hydrogel-based assay for short oligonucleotides from complex media, including larger interfering biomolecules.

## 2. Results and Discussion

**Figure 1:**
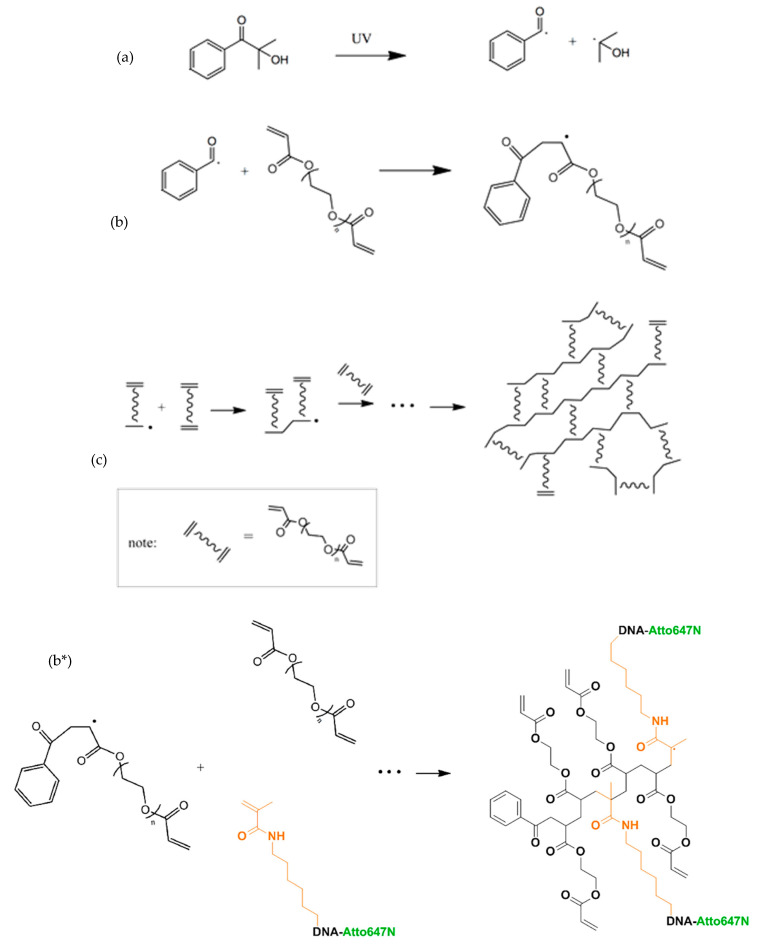
UV free radical photopolymerization: (**a**) Initiation mechanism, (**b**) Propagation mechanism, (**c**) Formation of a crosslinked polymer network (b*) UV free radical photopolymerization between PEGDA and methacrylate oligonucleotide.

The bulk hydrogels were synthesized using a UV free radical photopolymerization. The process involves three basic steps (Figure 1a). First, a free radical must be formed through the initiation step. For the PEGDA system used in this work, the initiator is DAROCUR1173, which forms a free radical under UV light (Figure 1a). The next step is known as propagation, where the free radical from the initiator transfers the radical to the carbon-carbon double bond in the acrylate functional group of a PEGDA molecule (Figure 1b). This step produces a second free radical species, which can go on to react with more PEGDA polymers, propagating the crosslink. The final step in the process of this polymerization is the termination, which occurs when two radical species meet and bonds form between them. A general reaction scheme for difunctional monomers in a radical polymerization forming a crosslinked network is reported (Figure 1c). In the case of DNA-PEG hydrogels, oligonucleotides are polymerized starting from methacrylate strands and treated in the same way as PEGDA hydrogels.

**Figure 2:**
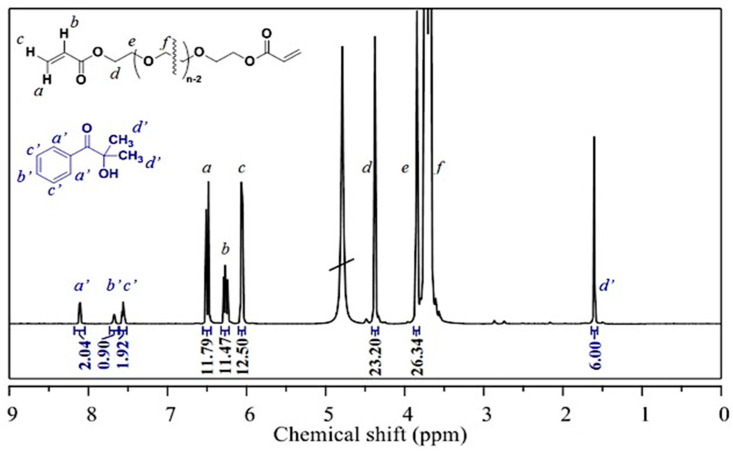
^1^H-NMR spectrum of PEGDA/darocur mixtures before polymerization.

In Figure 2, ^1^H-NMR spectra of PEGDA/darocur solution before polymerization is shown. Very intense peaks at 3.73 (*f*), 3.85 (*e*) and 4.38 (*d*) ppm are typical of PEG main chain methylene group, while peaks *a*, *b* and *c* in the olefinic region (6.0 to 6.6 ppm) refer to PEGDA terminals acrylic protons. Peaks in the aromatic region (*a’*, *b’* and *c’* from 7.5 to 8.2 ppm) and singlet at 1.45 ppm (*d’*) belong, respectively, to aromatic and methyl group protons of darocur. Broad peak at 4.65 ppm is the H_2_O residual signal. In Figure S2 is shown H^1^-NMR spectra of the PEGDA hydrogels soon after the crosslinking. The complete polymerization was confirmed by the disappearing of the PEGDA acrylic signals (region 6 to 6.6 ppm, See Supp Info Figure S1). In addition, the very broad peak from 4.5 to 3.5 ppm showed the transition of PEG chains from a very flexible situation (solution) to a less flexible state of crosslinked chains. Relative decrease of the DAROCUR 1173 peak intensities also confirmed that a large amount of the initiator was consumed in the polymerization reaction. The broad peak at 4.65 ppm is the H_2_O residual signal. One-dimensional ^1^H-NMR spectra of PEGDA/DAROCUR 1173 solutions were recorded before and after UV treatment, and all the comparisons of the different components are reported in supplementary information (Figure 2 and Figure S2).

### 2.1. Molecular Parameters of the Three-Dimensional Network

The swelling ratio and polymer volume fraction in the swollen state can be measured through an equilibrium swelling experiment [16]. Then the molecular weight and the mesh size can be obtained by a theoretical model for swelling of polymer hydrogels [27]. Indeed, the structure of hydrogels can be analyzed by the Flory–Rehner theory, subsequently modified by Peppas and Merrill, who developed a theoretical framework that has significant success in describing the hydrogel swelling behavior [13,21].

Cylindric PEGDA hydrogels of 1 cm height and 1 cm in diameter were prepared at different polymer concentration (10–15–20–25–30% *w*/*v*) to obtain various network structure with different molecular filter capability. Water content was experimentally measured by gravimetry (F_water_), and all fundamental swelling parameters derived (v_2,s_, Q, ¯;M_c_, ξ) for each preparation are summarized in Table 1.

First water content ranges from 91% up to 73% with the increasing of polymer concentration. Changing the PEGDA concentration, polymer volume fraction results in the range of 0.08–0.24. As expected, Q, ¯;M_c_, and ξ decreased with the increase of polymer concentration. In fact, the equilibrium swelling ratio decreased from 12.6 to 4.1 as the crosslinking density increased. The molecular weight between crosslinks decreased from 340 to 259 Da as the percent polymer fraction increased from 10 to 30% (*w*/*v*). Moreover, mesh size (ξ) linearly decreased with the increase of polymer fraction, ranging from 2.6 in the case of 10% up to 1.6 nm in the case of 30% (Figure S3). These results confirm that the polymer concentration permit to optimize the network density, as well as the mesh size, for the specific purpose.

### 2.2. NMR: Diffusion Studies

The NMR obtains information on the molecular mobility of an observed component. The advantage of this analysis is that it works in the absence of concentration gradients, studying instead the chaotic motion of molecules driven by intermolecular collisions [32]. Diffusion times over a large range, usually from a few milliseconds up to seconds, are available via PFG-NMR techniques and can be chosen arbitrarily by selecting the correct attenuation parameters [44]. The study of the probe diffusion on these short timescales, allows for obtaining much information on the microscopic structure of materials [32,42,43,44,45,47]. In particular, we are interested in measuring the water self-diffusion coefficient (D) that is directly related to the potential for water to leave a hydrogel [46,48,49,50].

DOSY experiments were used to study the diffusion of molecules, with different size, in hydrogel samples prepared with a PEGDA concentration of 10%, 15% and 20% (*w*/*v*). Firstly, we evaluated the water self-diffusion coefficient. The resulting measurement resulted was 24.55 × 10^−10^ m^2^/s (±0.11 × 10^−10^ m^2^/s), in agreement with literature data [51,52,53,54]. Furthermore, diffusion coefficient of solvent in the first hydration shell of hydrogels sample was calculated. Results indicated that water diffusion coefficient was affected by the PEGDA concentrations, as shown in Figure S7 (fitting curve reported in Figures S4–S6).

Moreover, several probes were properly chosen to simulate the behavior and diffusion of biological molecules. In particular, the smallest dextran (3 KDa) has hydrodynamic radius comparable with oligonucleotide (that represent molecule that we want to capture). On the other hand, 40 KDa dextran and blood proteins, such as albumin, have similar hydrodynamic radii and represent molecules that should be excluded from the network. In order to validate our choice, hydrodynamic radii of sulfo-rhodamine G and dextrans (3 KDa, 10 KDa, 40 KDa) were calculated using Einstein–Stokes equation. Results, reported in Table 2, showed that probes diffusion coefficients in D_2_O (D_0_) were in the range of 0.5 × 10^−10^–3.75 × 10^−10^ m^2^/s and their hydrodynamic radii had values between 3.9 and 0.6 nm. These molecules had a size comparable with the range between small oligonucleotides and large molecules. Afterwards, probes diffusion coefficients in bulk hydrogels (10%, 15%, 20% *w*/*v*) were calculated, as reported in Table 2. Diffusion coefficient of small molecules, such as sulforhodamine G (SR-G), should not be affected by a very large network structure and their values should be comparable with free diffusion of probe in water [3]. On the other hand, for high molecular weight molecules such as dextrans, we expect a decreasing trend of diffusion coefficient values from the water solution to the highest polymer percentage hydrogel.

**Figure 3:**
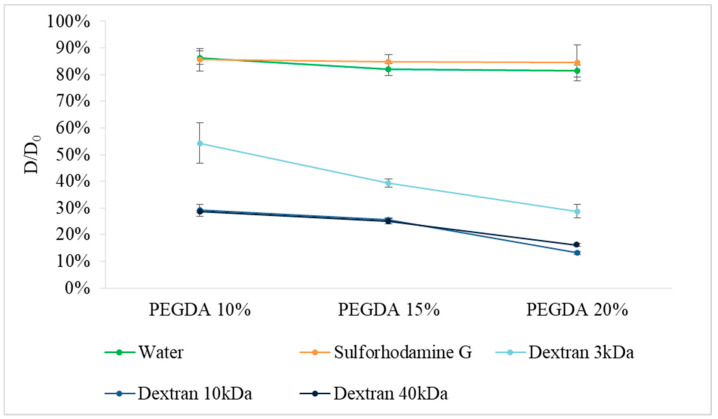
Effect of the polymer concentration on the reduction of diffusion coefficients (D/D_0_).

Moreover, the effect of the polymer concentration on the reduction of diffusion coefficients is highlighted in Figure 3, where D/D_0_ was plotted against polymer concentration. D/D_0_ values of water and sulforhodamine G, the smallest probe, were constant (~85%) for all PEGDA concentrations, however, in contrast, the dextran probe showed a significant mobility reduction, related with the polymer concentrations. In particular, for 3 KDa dextran, D/D_0_ decreased from 55% in the wider network, up to 30% in the tighter one. Instead, both 10 KDa and 40 KDa had a similar trend, between 30% and 15%. This confirms that the mobility of these bigger molecules is deeply affected by the polymer network.

NMR analyses show that hydrogels with different PEGDA concentrations are able to largely impair molecule trafficking because of their low diffusion coefficient. However, we can modulate the diffusion of the probe by modifying the mesh size of the network, changing the PEGDA concentrations. Dextran 3 KDa are able to diffuse through all the networks, while Dextrans 10 KDa and 40 KDa showed a very low mobility in the network with the largest molecular mesh.

### 2.3. Assay Set-Up

**Scheme 1:**
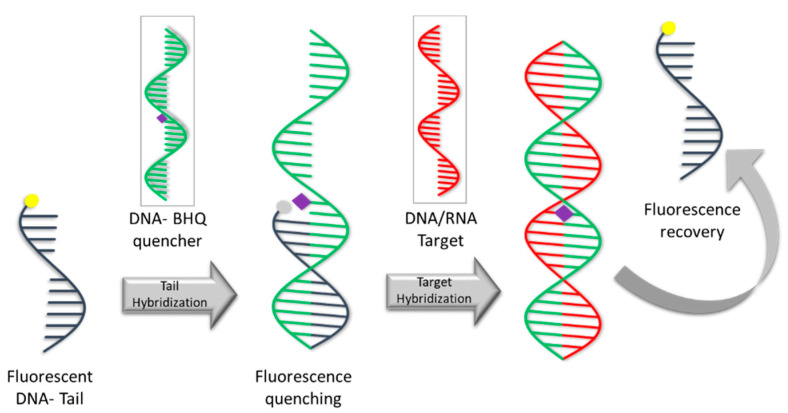
DNA-PEG hydrogels with antifouling and permselectivity toward high molecular weight interfering molecules. In the lower part of the scheme the oligonucleotide detection system implemented in the DNA-PEG hydrogels: fluorescent DNA-tail (T-DNA) immobilized in the hydrogel is partially hybridized with a longer complementary DNA-BHQ quenching strand; in presence of DNA or RNA target the displacement brings the formation of the hybridized DNA-BHQ/DNA or RNA target, with recovery of fluorescence inside the hydrogel (see Table S1 for the design based on thermodynamic parameters).

**Figure 4:**
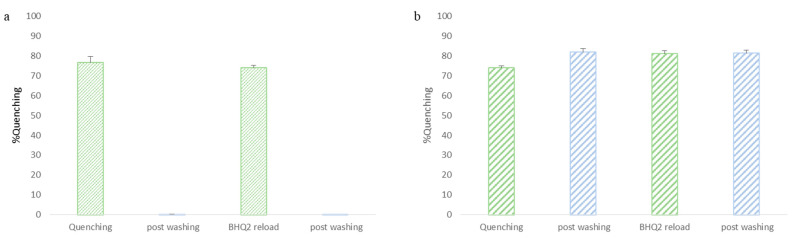
Quenching efficiency of ATTO-BHQ with, ¯;M_c_ 311 Da and mesh size 2.0 nm (PEGDA 20%): (**a**) and with, ¯;M_c_ 340 Da and mesh size 2.7 nm (PEGDA 10%); (**b**), by using 5 μM of BHQ strand.

Preliminary studies for the detection assay optimization were performed using a probe scheme already tested in previous work by our group [55,56,57,58]. An overview of probe mechanism is reported in Scheme 1 (lower part), where fluorescent T-DNA is a short fluorescent DNA tail (12 nt), labelled at 5′ end with ATTO 647N and modified at 3′ with methacrylamide spacer to allow its covalent binding with polymer network. A Quencher strand (21 nt), internally modified with a Black Hole Quencher (BHQ), was used as probe diffusion. When the tail hybridizes, the BHQ comes in close proximity with the fluorophore and fluorescence quenching occurs (for sequence and thermodynamic parameters see Sup. Info). Here we probed the quenching effect due to the partial hybridization of the T-DNA, immobilized in the hydrogel, and the quencher strand BHQ. After an appropriate optimization, we tested the efficiency of quenching on DNA-PEG hydrogels obtained with 5 μM T-DNA co-polymerized (see Figure S8). We reported the fluorescence intensity for the upper and lower polymer concentration (20% and 10% (*w*/*v*)). We added BHQ strand and repeated the analysis (Figure S9). Comparing values of quenching percentage for the two different polymer concentrations, we obtained a similar quenching (~75%). After a washing of the bulk with PBS buffer, we report a high recovery of fluorescence (above 95%) for PEGDA 20% bulk. In that case, the quenching was only transient even after a second repetition of the quenching step. In this case, in fact, the BHQ strand diffuse in the bulk and comes in proximity with the fluorophore. This is the case of dynamic quenching (Figure 4a). Here, the correct hybridization does not occur to form a stable double strand, while the quenching strand comes near enough to collide with fluorophore with a consequent fluorescence decrement. By contrast, in PEGDA 10%, the network was sufficiently wider to allow the formation of stable complex between oligonucleotide strands, so that the tail and quencher came in proximity. In this case, quenching effect is shown and the partial hybridization occurred to form a stable duplex even after several washes (Figure 4b). The formation of the DNA duplex implies that the two interacting molecules (T-DNA and BHQ strand, in this case) are in the correct orientation to form the hydrogen bonds between the complementary bases. The tight mesh size would provide a limited space and a stiffening of the structure. Both effects could, in turn, limit the degree of freedom of the immobilized molecules to explore the space and assume the right orientation for the formation of the hydrogen bonds.

**Figure 5:**
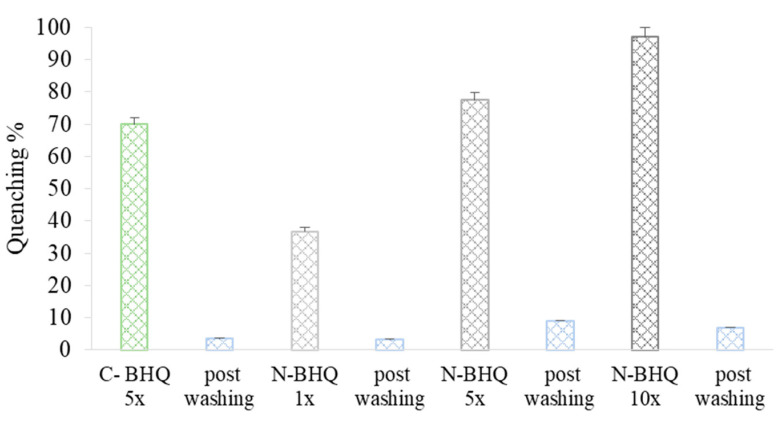
Quenching efficiency with complementary strand (C-BHQ) in green and non-complementary strand (N-BHQ) in grey at different concentrations.

To prove such hypothesis, we tested 50% DNA-PEG hydrogels using both complementary (C) and non-complementary (N) BHQ probes. As reported in Figure 5, after the addition of C-BHQ, we obtained a ~70% quenching that strongly decreased at ~3% after washing. This result is also confirmed by adding a noncomplementary strand N-BHQ. In this case, despite the increase of quenching with N-BHQ amount up to 10-fold, we found a huge recovery of the fluorescence after washings. Therefore, we can conclude that both tight mesh size and high concentration of T-DNA inside the bulk do not allow for an effective duplex hybridization, even at high BHQ-strand concentrations.

We also investigated the effect of the mesh size and density of functionalization with T-DNA by following the kinetic of hybridization of the BHQ strand over a long time (120 h). All tests were carried out with 1 μM and 5 μM F-DNA-Tail with BHQ strand 1× and 5× with respect to the nominal T-DNA density.

**Figure 6:**
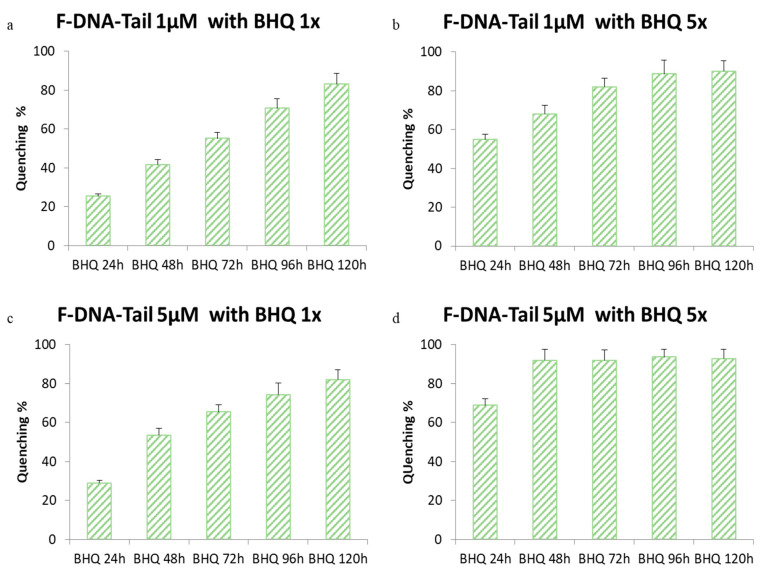
Quenching kinetic in PEGDA 10% with several oligonucleotides concentrations: (**a**) F-DNA-Tail 1 μM-BHQ 1×; (**b**) F-DNA-Tail 1 μM-BHQ 5×; (**c**) F-DNA-Tail 5 μM-BHQ 1×; (**d**) F-DNA-Tail 5 μM-BHQ 5×.

Regarding PEGDA 10%, Figure 6 shows that quenching percentage increased with the increasing of both T-DNA and BHQ concentrations. In the case of T-DNA 1 μM with BHQ in 1× (Figure 6a), we see a linear increase of quenching up to 80% after 120 h. Further increase of quenching strand (BHQ 5×) (Figure 6b) brings a quenching efficiency of about 80% after 72 h. After 120 h, the efficiency of quenching was above 90%. Increasing the concentration of T-DNA at 5 μM brings a slight acceleration of quenching for the lower BHQ (Figure 6c), while the increase to BHQ 5× (Figure 6d) produces a quenching efficiency above 90% after only 48 h.

**Figure 7:**
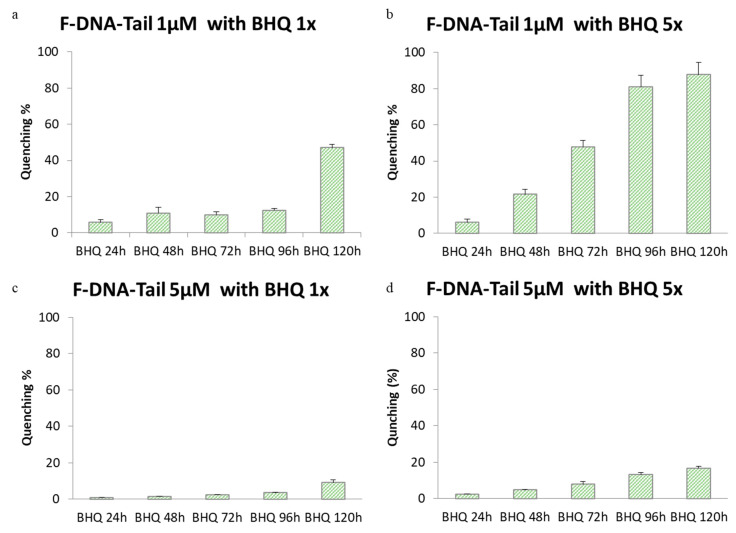
Quenching kinetic in PEGDA 15% with several oligonucleotides concentrations: (**a**) F-DNA-Tail 1 μM-BHQ 1×; (**b**) F-DNA-Tail 1 μM-BHQ 5×; (**c**) F-DNA-Tail 5 μM-BHQ 1×; (**d**) F-DNA-Tail 5 μM-BHQ 5×.

On the other hand, for PEGDA 15%, only in the case of T-DNA 5 μM and BHQ 1× was it possible to reach, after 96 h, an 80% quenching (Figure 7). In fact, at constant T-DNA, a lower concentration BHQ 1× was not enough to provide a good quenching, while an increase of T-DNA hinders the effective hybridization.

**Figure 8:**
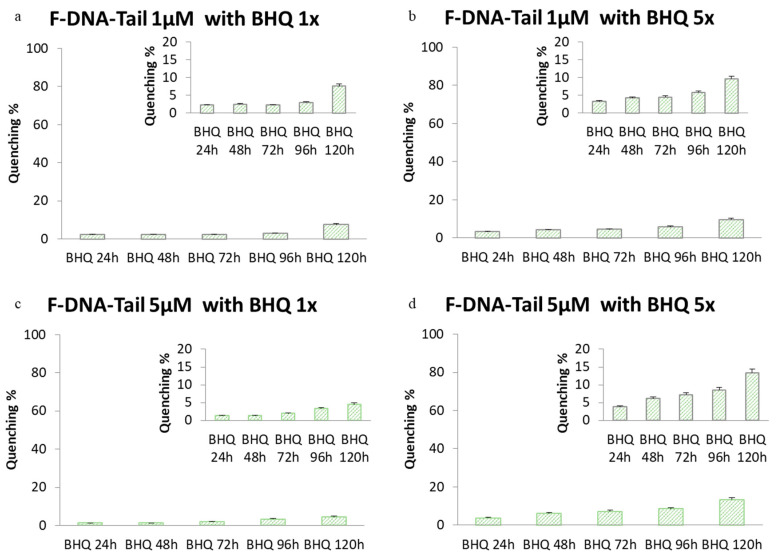
Quenching kinetic in PEGDA 20% with several oligonucleotides concentrations: (**a**) F-DNA-Tail 1 μM-BHQ 1×; (**b**) F-DNA-Tail 1 μM-BHQ 5×; (**c**) F-DNA-Tail 5 μM-BHQ 1×; (**d**) T-DNA 5 μM-BHQ 5×.

Finally, for PEGDA 20% we reached very low values of quenching for all sample preparations (Figure 8).

**Figure 9:**
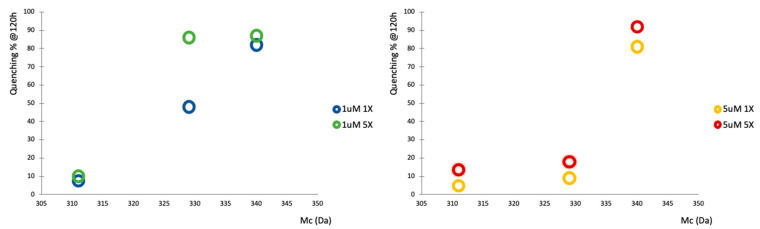
Comparison of quenching after 120 h for 1 μM and 5 μM DNA-PEG hydrogels with different crosslinking densities (¯;M_c_).

In this case the efficiency of quenching does not reach more than 10%, and the effect of mesh size and density of functionalization is more evident in the obstruction of free diffusion of quenching molecules. Moreover, the decrease of quenching percentage post-washing confirmed the inefficient hybridization (data not shown). Overall, such observations are useful to define the behavior of functionalized hydrogels for the direct capture of oligonucleotides. Figure 9 shows the quenching efficiency toward the density of crosslinking defined as molecular weight between crosslinks (M_c_). After longer times (120 h), we can see how oligonucleotides can pass through the material only when crosslinks are large enough.

**Figure 10:**
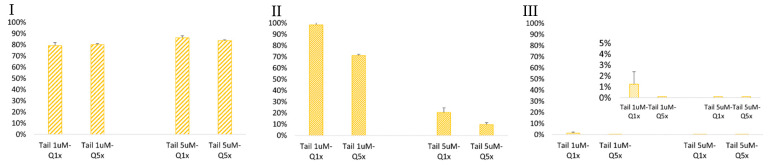
Fluorescence recovery after displacement with 10X Target-DNA in DNA-PEG hydrogel 10% (**I**)—15% (**II**) and 20% (**III**) with Tail concentrations of 1 μM and 5 μM.

To provide insight on the possibility to use such design for a displacement assay, we proceed to test the capability of DNA-PEG hydrogels to serve as selective sponges. We added the target to each polymer concentrations with density of functionalization of 1 μM and 5 μM. The analysis was performed for all bulk hydrogels tested for quenching. All tests were carried out with Target concentration 10× with respect of T-DNA immobilized and the fluorescence recovery was measured, taking as reference the quenched level (Figure 10).

At first glance, the 10% DNA-PEG hydrogels produced a high recovery of fluorescence in all cases. In the case of 15%, a drop in recovery of fluorescence is observed at higher density of functionalization, while for 20% DNA-PEG hydrogels the recovery is very poor.

Overall, these results confirm that higher polymer concentration produce a crowding effect that reduce molecular mobility inside the hydrogel and, at the same time, a stiffening of the network that reduces the flexibility of the capturing DNA tail. Polymer concentrations higher than 15% of PEGDA were not suitable for probe diffusion, resulting in slow down even for small molecules (below 3 KDa). In this case, it was observed that the recognition in 3D resulted in a more effective reduction in polymer concentration and density of functionalization. The results presented make the recognition in 3D a complex balance between the mesh size, the stiffness of the substrate and the density of functionalization. As observed here the reduction of mesh size provides the space for the diffusion of small oligonucleotides even though the reduced mobility of the capture molecules does not allow for proper hybridization.

## 3. Conclusions

In this paper, we focused on the development of PEGDA-based bulk hydrogels for perm-selective detection of small DNA oligonucleotides (21 bp). Swelling characterization showed the possibility of modulating mesh size by changing the polymer concentration. This is the case to achieve filtering capability of the 3D hydrogels with regards of high molecular weight molecules.

NMR analysis confirmed the opportunity to use hydrogels as molecular filter. In particular, 10% and 15% polymer concentrations resulted suitable to be used as molecular filters with cut-off of radius of 2.4 nm comparable to the mesh size. In fact, these networks allowed the diffusion of molecules with size comparable with our probe (oligonucleotides) and excluded larger molecules with size similar to proteins and antibody.

Moreover, fluorometric analysis gave us important information regarding the ratio between polymer and oligonucleotides concentrations to obtain an efficient hybridization. In particular, 10% and 15% PEGDA concentrations showed a suitable network for both oligonucleotide diffusion and effective hybridization. In addition, results showed that for 10% of polymer we can obtain a good quenching percentage also for the highest oligo concentration. On the other hands, for 15% of polymer, we cannot use oligonucleotide concentration higher than 1 μM, because of crowding effect.

In conclusion, our results showed the possibility to obtain functionalized and engineered materials, with a tunable network, that allow to combine both molecular filter and capture element capabilities. In this way, we can improve these materials to simplify the detection analysis of several target.

## 4. Materials and Methods

### 4.1. Materials and Reagents

Poly(ethylene glycol)diacrylate (PEGDA-700 Da), Deuterium oxide (D_2_O), Sulforhodamine G, Ethanol and Diethyl ether were purchased from Sigma-Aldrich (St. Gallen, CH) and used as received. Crosslinking reagent 2-Hydroxy-2-methylpropiophenone (DAROCUR 1173, CIBA, Basel, Switzerland) was provided from Ciba. Texas Red labelled dextrans with different molecular weight (3 KDa, 10 KDa and 40 KDa) were supplied by Thermo Fisher Scientific (Milano, Italy. DNA oligonucleotides were purchased from Metabion with HPLC (Monaco, Germany) purification. NMR tubes, properly designed for gel samples, were supplied by New Era Enterprises, Inc. and OptiPlate-96 F HB were purchased by PerkinElmer.

### 4.2. Synthesis of Bulk-Hydrogel

The hydrogels used here were synthesized by UV free radical polymerization using poly(ethylene glycol) diacrylate (PEGDA, 700 Da) at different concentrations in Milli-Q water and DAROCUR 1173 used as initiator [57]. Each hydrogel sample was prepared dissolving photoinitiator in 10 mL of polymer solution, obtaining an initiator final concentration of ~0.1% *v*/*v*. The reaction mixture was strongly mixed and then purged with high purity nitrogen for 5 min to remove the excess of oxygen that may interfere with the free radical polymerization. The reaction mixture was then carried out in 10 mL test tubes and illuminated with UV lamp (λ = 365 nm and power-lamp = 10 W) for 5 min leading to complete polymerization. For NMR studies, we reduced the total reaction volume to 1 mL of deuterated water. Polymerization was performed directly in special NMR tubes designed for gel samples. Hydrogel samples were than expelled from the NMR tube and put in a solution of D_2_O for the next 24 h to remove all the unreacted materials. Hydrogels were dried in oven at 30 °C for few hours, swelled with 1 mL of probe solution and finally put in the NMR tube for the measurement. Different probes, sulforhodamine G and several dextrans (3 KDa, 10 KDa and 40 KDa) were dissolved in D_2_O to reach a final concentration of 1 mg/mL. Moreover, functionalized bulk were synthesized using UV free radical photopolymerization between PEGDA and oligonucleotide, properly modified with methacrylamide moieties. Propagation step of the reaction between polymer and methacrylate oligonucleotide is highlighted in Figure 1b*.

Several recipes with different PEGDA and oligonucleotide concentrations were prepared and tested. The mixture was composed of PEGDA (MW 700 Da) at different concentrations (10–15–20% *w*/*v*) in buffer solution, DAROCUR 1173 as initiator and fluorescent oligonucleotide methacrylate (F-DNA-Tail) at different concentrations (1–5 μM). Our buffer solution was obtained adding 1 × PBS and NaCl 200 mM in Milli-Q water. The reaction mixture was strongly mixed and then purged with high purity nitrogen for 5 min to remove the excess of oxygen that may interfere with the free radical polymerization. Then, 100 μL of the mixture was carried out in 96-optiplate, illuminated with the same UV lamp used before, for 5 min leading to complete polymerization.

### 4.3. Bulk-Hydrogel Characterizations

Several characterizations, attained due to NMR and fluorimeter measurements, were accomplished on different bulk samples to define the structural and diffusion properties of these materials in both PEG-bulk and functionalized hydrogels [58].

The swelling characterization of hydrogels was carried out applying the equilibrium swelling theory, also known as Flory–Rehner theory. The theory is based on the following equation that allows to calculate the molecular weight between crosslinks that represent a measure of the degree of crosslinking of the polymer. Obviously, due to the random nature of the polymerization process itself, only average values of ¯;M_c_ can be calculated with Equation (1).

(1) $$\frac{1}{{\bar{M}}_{c}}=\frac{2}{{\bar{M}}_{n}}-\frac{\left(\frac{\bar{v}}{V_{1}}\right)\left[\ln\left(1-v_{2,s}\right)+v_{2,s}+\chi_{1}v_{2,s}^{2}\right]}{\left(v_{2,s}^{1/3}-\frac{v_{2,s}}{2}\right)}$$

In this equation ¯;M_n_ is the molecular weight of the polymer chains prepared in the absence of a crosslinking agent, v_2,s_ is the polymer fraction in the swollen state, χ_1,2_ is the polymer-solvent interaction parameter, ¯;ν is the specific volume of the polymer, and V_1_ is the molar volume of water.

To apply this equation, several parameters are needed: interaction parameter PEG/water, χ = 0.426 [43]; specific volume, v = 0.91 mL/g; molar volume of water, V1= 18.1 mL/mol [43]; uncrosslinked molecular weight of PEG, M_n_ = 700 Da [46,49,50]. Polymer volume fraction in the swollen state (v_2,s_) characterizes how well the polymer absorbs water. It can be expressed as the ratio of the volume of dry polymer, V_d_, to the volume of the swollen polymer gel, V_s_, and is the reciprocal of the degree of swelling, Q [16,46]. Here we calculated v_2,s_ using Equation (2) above.

(2) $$v_{2,s}=\frac{V_{d}}{V_{s}}=\frac{1}{Q}=\frac{1}{1+\frac{\rho_{polymer}}{\rho_{solvent}}\left(\frac{m_{s}}{m_{d}}-1\right)}$$

Masses in both conditions were determined as follow: once polymerized, hydrogels were removed from the glass tubes, uniformly cut into samples of short cylindrical shape (1 cm height, 1 cm diameter) and then immersed in an excess of Milli-Q water in order to remove the unreacted reagents. Water was changed several times for the next 24 h and samples were then swollen overnight in water at room temperature. Then cylindrical parts were gently wiped with a filter paper and weighed to obtain the mass of swollen hydrogel (m_s_). For dried measurement, in order to obtain a total dehydration, hydrogels were properly ground and lyophilized overnight, so the mass of dry samples were recovered (m_d_). Therefore, we were able to determine hydrogel volume in both swollen and dry state applying Equation (2). Moreover, we evaluated the water content of the gels, which represent the mass fraction of water (F_water_) in the swollen condition using Equation (3)

(3) $$F_{\mathrm{water}}=\frac{m_{\mathrm{swollen}}-m_{\mathrm{dry}}}{m_{\mathrm{swollen}}}$$

Once M_c_ was calculated, the mesh size (ξ) can be determined from the following equation [15]:

(4) $$\xi =v_{2,s}^{-1/3}{\left({\bar{r}}_{0}^{2}\right)}^{1/2}\;\;$$

where ${\bar{r}}_{0}^{2}$ is the value of the end-to-end distance of PEG chains in the unperturbed state and it is calculated applying Equation (5):

(5) $${\left(r_{0}^{2}\right)}^{1/2}=C_{n}{\mathrm{Nl}}^{2}$$

where C_n_ is the Flory characteristic ratio, l is the length of each link and N, the number of links in the chain, is given by Equation (6):

(6) $$N=\frac{2{\bar{M}}_{c}}{M_{r}}$$

where M_r_ is the molecular weight of the repeating unit of the polymer.

*NMR analysis* was performed using an Agilent 600 MHz (14 Tesla) spectrometer equipped with a DD2 console. ^1^H-1D-NMR spectra were recorded at 300 K with 64 scans to obtain a good signal-to-noise ratio. Diffusion spectra were recorded at 300 K using a Dbppste (DOSY bipolar pulse pair stimulated echo) pulse sequence [50]. An array of 15 pulse field gradient values from 0:98 to 63:7 Gauss/cm were used with a gradient pulse length (δ) of 2 ms. Diffusion delay (Δ) in ms was optimized for each sample to obtain a ratio of 1:0.2 between I(Gmin) and I(Gmax), where I(Gmin) is the intensity of the NMR peak at the minimum gradient value, and I(Gmax) is the intensity of the NMR peak at the maximum gradient value. Diffusion coefficients were calculated using the formula reported in Equation (7):

(7) $$I\left(\delta ,\Delta \right)=I_{0}exp\left[-D\gamma^{2}g^{2}{}^{2}\left(\Delta -\frac{\delta }{3}\right)\right]$$

where I and I_0_ are the spin echo amplitudes in the presence and in the absence of a magnetic field gradient pulse, respectively; γ is the gyromagnetic ratio of the given nucleus, Hz/T, and g is the magnetic gradient, T/m. NMR data were processed and analyzed using VNMRJ4 software.

Fluorescence analysis. The best concentration of both polymer and oligonucleotide was evaluated and the effective hybridization of Fluorescent-DNA-Tail and BHQ-strand checked. Fluorescence spectra of fixed number of labelled oligonucleotides were collected in a 1 cm path length cuvette with a Horiba JobinYvon model FluoroMax-4 fluorometer equipped with a Peltier temperature controller. The conjugated and unconjugated sequences were excited at 647 nm with a slit width of 5 nm, and emission spectra were collected from 667 to 750 nm with a slit width of 5 nm. Then, hydrogel bulks were synthetized into optiplate 96 F and analyzed by the means of a 2300 EnSpire multilabel reader (Perkinelmer, Waltham, MA, USA). Fluorescence was measured from the top of the plate, with excitation wavelength of 647 nm and emission wavelength at 667 nm, height 9mm and 500 number of flashes.

## Figures and Tables

**Table 1:** Properties of bulk hydrogels of PEGDA. All values were calculated from averaged measured values.

	PEGDA 10%	PEGDA 15%	PEGDA 20%	PEGDA 25%	PEGDA 30%
**F_water_**	91.2% ± 0.1%	87.2% ± 0.1%	82.7% ± 0.1%	78.2% ± 0.1%	73.5% ± 0.1%
**v_2,s_** (10^−3^)	79.2 ± 0.9	116.2 ± 1.8	157.5 ± 0.5	199.3 ± 0. 5	243.4 ± 0.4
**Q**	12.63 ± 0.14	8.61 ± 0.14	6.349 ± 0.018	5.017 ± 0.012	4.109 ± 0.008
**¯;M_c_** (Da)	340.43 ± 0.21	329.35 ± 0.66	311.62 ± 0.23	288.24 ± 0.30	259.06 ± 0.31
**ξ** (nm)	2.7 ± 0.1	2.3 ± 0.1	2.0 ± 0.1	1.8 ± 0.1	1.6 ± 0.1

**Table 2:** Self-diffusion (D_0_ [10^−10^ m^2^/s]), hydrodynamic radii (R_0_ [nm]) and diffusion coefficient (10^−10^ m^2^/s) of water and different probes in both water and PEGDA hydrogels.

	D_0_	R_0_	D PEGDA 10%	D PEGDA 15%	DPEGDA 20%
Water	24.55 ± 0.11	0.09 ± 0.01	18.84 ± 0.03	16.05 ± 0.02	15.08 ± 0.13
Sulforhodamine G	3.67 ± 0.06	0.61 ± 0.01	3.14 ± 0.15	3.11 ± 009	3.10 ± 0.24
Dextran 3 kDa	1.60 ± 0.03	1.34 ± 0.01	0.87 ± 0.12	0.63 ± 0.02	0.46 ± 0.04
Dextran 10 kDa	0.91 ± 0.01	2.41 ± 0.01	0.27 ± 0.02	0.234 ± 0.001	0.120 ± 0.005
Dextran 40 kDa	0.557 ± 0.008	3.92 ± 0.03	0.160 ± 0.001	0.140 ± 0.008	0.090 ± 0.008

## Supplementary Materials

The following are available online at https://www.mdpi.com/article/10.3390/gels7030090/s1, Figure S1: 1H-NMR spectra of individual components DAROCUR 1173 (IV) and PEGDA (III) and mixture pre (II) and post-polymerization (I); Figure S2: 1H-NMR spectrum of PEGDA/darocur mixtures after polymerization; Figure S3: Mesh size values for different bulk-PEGDA concentrations; Figure S4: NMR-DOSY for water diffusion in PEGDA 10%; Figure S5: NMR-DOSY for water diffusion in PEGDA 15%; Figure S6: NMR-DOSY for water diffusion in PEGDA 20%; Figure S7: 2D DOSY of water (4.645 ppm) in hydrogels bulk with different PEGDA concentrations (20% in blue, 15% in red and 10% in black). Table S1: Sequence and thermodynamic parameters of the DNA probes used in this study. Figure S8: Quenching percentage in solution for different oligonucleotide concentrations; Figure S9: Fluorescence intensity of ATTO-BHQ in PEGDA 20% (a) and 10% (b).

## References

[1] Pluen A., Netti P., Jain R.K., Berk D.A. (1999). Diffusion of Macromolecules in Agarose Gels: Comparison of Linear and Globular Configurations. Biophys. J..

[2] Liu D.E., Kotsmar C., Nguyen F., Sells T., Taylor N.O., Prausnitz J.M., Radke C.J. (2013). Macromolecule Sorption and Diffusion in HEMA/MAA Hydrogels. Ind. Eng. Chem. Res..

[3] Cheng Y., Prud’Homme R.K., Thomas J.L. (2002). Diffusion of Mesoscopic Probes in Aqueous Polymer Solutions Measured by Fluorescence Recovery after Photobleaching. Macromolecules.

[4] Sharma B., Thakur S., Mamba G., Prateek, Gupta R.K., Gupta V.K., Thakur V.K. (2021). Titania modified gum tragacanth based hydrogel nanocomposite for water remediation. J. Environ. Chem. Eng..

[5] Dehshahri A., Kumar A., Madamsetty V., Uzieliene I., Tavakol S., Azedi F., Fekri H., Zarrabi A., Mohammadinejad R., Thakur V. (2020). New Horizons in Hydrogels for Methotrexate Delivery. Gels.

[6] Hoffman A.S. (2012). Hydrogels for biomedical applications. Adv. Drug Deliv. Rev..

[7] Peppas N.A., Moynihan H.J., Lucht L.M. (1985). The structure of highly crosslinked poly (2-hydroxyethyl methacrylate) hy-drogels. J. Biomed. Mater. Res. Part A.

[8] Anseth K.S., Bowman C., Brannon-Peppas L. (1996). Mechanical properties of hydrogels and their experimental determination. Biomaterials.

[9] D’Errico G., De Lellis M., Mangiapia G., Tedeschi A., Ortona O., Fusco S., Borzacchiello A., Ambrosio L. (2008). Structural and Mechanical Properties of UV-Photo-Cross-Linked Poly(N-vinyl-2-pyrrolidone) Hydrogels. Biomacromolecules.

[10] Hoffman A.S., Schmer G., Harris C., Kraft W.G. (1972). Covalent binding of biomolecules to radiation-grafted hydrogels on inert polymer surfaces. ASAIO J..

[11] Zalipsky S., Harris J.M. (1997). Introduction to Chemistry and Biological Applications of Poly(ethylene glycol). ACS Symp. Ser..

[12] Mohammadinejad R., Maleki H., Larrañeta E., Fajardo A.R., Nik A.B., Shavandi A., Sheikhi A., Ghorbanpour M., Farokhi M., Govindh P. (2019). Status and future scope of plant-based green hydrogels in biomedical engineering. Appl. Mater. Today.

[13] Peppas N.A., Merrill E.W. (1977). Crosslinked poly(vinyl alcohol) hydrogels as swollen elastic networks. J. Appl. Polym. Sci..

[14] Peppas N.A., Huang Y., Torres-Lugo M., Ward J.H., Zhang J. (2000). Physicochemical Foundations and Structural Design of Hydrogels in Medicine and Biology. Annu. Rev. Biomed. Eng..

[15] Peppas N.A., Keys K.B., Torres-Lugo M., Lowman A.M. (1999). Poly (ethylene glycol)-containing hydrogels in drug delivery. J. Control. Release.

[16] Peppas N.A., Hilt J.Z., Khademhosseini A., Langer R. (2006). Hydrogels in Biology and Medicine: From Molecular Principles to Bionanotechnology. Adv. Mater..

[17] Yoo K., Murphy S., Skardal A. (2021). A Rapid Crosslinkable Maleimide-Modified Hyaluronic Acid and Gelatin Hydrogel Delivery System for Regenerative Applications. Gels.

[18] Kopecek J. (2009). Hydrogels: From soft contact lenses and implants to self-assembled nanomaterials. J. Polym. Sci. Part A Polym. Chem..

[19] Yasuda H., Peterlin A., Colton C.K., Smith K.A., Merrill E.W. (1969). Permeability of solutes through hydrated polymer membranes. Part III. Theoretical background for the selectivity of dialysis membranes. Die Makromol. Chem..

[20] Peppas N., Bures P., Leobandung W., Ichikawa H. (2000). Hydrogels in pharmaceutical formulations. Eur. J. Pharm. Biopharm..

[21] Peppas N.A., Khare A.R. (1993). Preparation, structure and diffusional behavior of hydrogels in controlled release. Adv. Drug Deliv. Rev..

[22] Bryant S.J., Anseth K.S. (2002). Hydrogel properties influence ECM production by chondrocytes photoencapsulated in poly(ethylene glycol) hydrogels. J. Biomed. Mater. Res..

[23] Amsden B. (1998). Solute diffusion in hydrogels.: An examination of the retardation effect. Polym. Gels Netw..

[24] Farnan D., Frey D., Horváth C. (2002). Surface and pore diffusion in macroporous and gel-filled gigaporous stationary phases for protein chromatography. J. Chromatogr. A.

[25] Farnan D., Frey D., Horváth C. (1997). Intraparticle Mass Transfer in High-Speed Chromatography of Proteins. Biotechnol. Prog..

[26] Peng C.-C., Kim J., Chauhan A. (2010). Extended delivery of hydrophilic drugs from silicone-hydrogel contact lenses containing Vitamin E diffusion barriers. Biomaterials.

[27] Lewus R.K., Carta G. (1999). Protein diffusion in charged polyacrylamide gels. Visualization and analysis. J. Chromatogr. A.

[28] Lewus R.K., Carta G. (2001). Protein transport in constrained anionic hydrogels: Diffusion and boundary-layer mass transfer. Ind. Eng. Chem. Res..

[29] Russell S.M., Belcher E.B., Carta G. (2003). Protein partitioning and transport in supported cationic acrylamide-based hydrogels. AIChE J..

[30] Russell S.M., Carta G. (2005). Multicomponent protein adsorption in supported cationic polyacrylamide hydrogels. AIChE J..

[31] Flory P.J. (1953). Principles of Polymer Chemistry.

[32] Wallace M., Adams D.J., Iggo J.A. (2013). Analysis of the mesh size in a supramolecular hydrogel by PFG-NMR spectroscopy. Soft Matter.

[33] Sun J., Lyles B.F., Yu K.H., Weddell J., Pople J., Hetzer M., De Kee D., Russo P.S. (2008). Diffusion of Dextran Probes in a Self-Assembled Fibrous Gel Composed of Two-Dimensional Arborols. J. Phys. Chem. B.

[34] Branco M.C., Pochan D.J., Wagner N.J., Schneider J.P. (2009). Macromolecular diffusion and release from self-assembled β-hairpin peptide hydrogels. Biomaterials.

[35] Cohen Y., Avram L., Frish L. (2005). Diffusion NMR spectroscopy in supramolecular and combinatorial chemistry: An old parameter—new insights. Angew. Chem. Int. Ed..

[36] Cao S., Fu X., Wang N., Wang H., Yang Y. (2008). Release behavior of salicylic acid in supramolecular hydrogels formed by l-phenylalanine derivatives as hydrogelator. Int. J. Pharm..

[37] Scalettar B.A., Hearst J.E., Klein M.P. (1989). FRAP and FCS studies of self-diffusion and mutual diffusion in entangled DNA solutions. Macromolecules.

[38] Watanabe N., Li X., Shibayama M. (2017). Probe Diffusion during Sol–Gel Transition of a Radical Polymerization System Using Isorefractive Dynamic Light Scattering. Macromolecules.

[39] Fatin-Rouge N., Starchev K., Buffle J. (2004). Size Effects on Diffusion Processes within Agarose Gels. Biophys. J..

[40] Banks D.S., Tressler C., Peters R.D., Höfling F., Fradin C. (2016). Characterizing anomalous diffusion in crowded polymer solutions and gels over five decades in time with variable-lengthscale fluorescence correlation spectroscopy. Soft Matter.

[41] Colsenet R., Söderman O., Mariette F. (2006). Pulsed Field Gradient NMR Study of Poly(ethylene glycol) Diffusion in Whey Protein Solutions and Gels. Macromolecules.

[42] Gao P., Fagerness P.E. (1995). Diffusion in HPMC Gels. I. Determination of Drug and Water Diffusivity by Pulsed-Field-Gradient Spin-Echo NMR. Pharm. Res..

[43] Kwak S., LaFleur M. (2003). Self-diffusion of macromolecules and macroassemblies in curdlan gels as examined by PFG-SE NMR technique. Colloids Surf. A Physicochem. Eng. Asp..

[44] Rashid H., Golitsyn Y., Bilal M., Mäder K., Reichert D., Kressler J. (2021). Polymer Networks Synthesized from Poly(Sorbitol Adipate) and Functionalized Poly(Ethylene Glycol). Gels.

[45] Gagnon M.-A., LaFleur M. (2010). Comparison between nuclear magnetic resonance profiling and the source/sink approach for characterizing drug diffusion in hydrogel matrices. Pharm. Dev. Technol..

[46] Kärger J. (1985). NMR self-diffusion studies in heterogeneous systems. Adv. Colloid Interface Sci..

[47] Gagnon M.-A., Lafleur M. (2009). Self-diffusion and mutual diffusion of small molecules in high-set curdlan hydrogels studied by 31P NMR. J. Phys. Chem. B.

[48] Escuder B., LLusar M., Miravet J.F. (2006). Insight on the NMR study of supramolecular gels and its application to monitor molecular recognition on self-assembled fibers. J. Org. Chem..

[49] Shapiro Y.E. (2011). Structure and dynamics of hydrogels and organogels: An NMR spectroscopy approach. Prog. Polym. Sci..

[50] McConville P., Pope J. (2000). A comparison of water binding and mobility in contact lens hydrogels from NMR measurements of the water self-diffusion coefficient. Polymer.

[51] Zustiak S.P., Leach J.B. (2010). Hydrolytically Degradable Poly(Ethylene Glycol) Hydrogel Scaffolds with Tunable Degradation and Mechanical Properties. Biomacromolecules.

[52] Thakur A., Wanchoo R., Singh P. (2011). Structural parameters and swelling behavior of pH sensitive poly (acrylamide-co-acrylic acid) hydrogels. Chem. Biochem. Eng. Q..

[53] Johnson C.S. (1999). Diffusion ordered nuclear magnetic resonance spectroscopy: Principles and applications. Prog. Nucl. Magn. Reson. Spectrosc..

[54] Holz M., Heil S.R., Sacco A. (2000). Temperature-dependent self-diffusion coefficients of water and six selected molecular liquids for calibration in accurate 1H NMR PFG measurements. Phys. Chem. Chem. Phys..

[55] Causa F., Aliberti A., Cusano A.M., Battista E., Netti P. (2015). Supramolecular Spectrally Encoded Microgels with Double Strand Probes for Absolute and Direct miRNA Fluorescence Detection at High Sensitivity. J. Am. Chem. Soc..

[56] Battista E., Mazzarotta A., Causa F., Cusano A.M., Netti P. (2016). Core—shell microgels with controlled structural properties. Polym. Int..

[57] Celetti G., Di Natale C., Causa F., Battista E., Netti P.A. (2016). Functionalized poly(ethylene glycol) diacrylate microgels by microfluidics: In situ peptide encapsulation for in serum selective protein detection. Colloids Surf. B Biointerfaces.

[58] Battista E., Causa F., Netti P.A. (2017). Bioengineering Microgels and Hydrogel Microparticles for Sensing Biomolecular Targets. Gels.

