# Supplementary material for: Small Oligonucleotides Detection in Three-Dimensional Polymer Network of DNA-PEG Hydrogels
Source: Gels. 2021 Jul 12;7(3):90. doi: 10.3390/gels7030090 (PMC8293047; doi:10.3390/gels7030090)
Supplement: Supplementary file 1 [file gels-07-00090-s001.zip › gels-1264671-supplementary.pdf]

Alessia Mazzarotta[1], Tania Mariastella Caputo[1], Luca Raiola[1], Edmondo Battista[2], Filippo Causa[1,2,3], Paolo Antonio Netti[1,2,3]

[1] *Center for Advanced Biomaterials for Healthcare@CRIB, Istituto Italiano di Tecnologia (IIT), Largo Barsanti e Matteucci 53, 80125 Naples, Italy*

[2] *InterdisciplinaryResearch Centre on Biomaterials (CRIB), Università degli Studi di Napoli "Federico II", Piazzale Tecchio 80, 80125 Naples, Italy*

[3] *Dipartimento di Ingegneria Chimica del Materiali e della Produzione Industriale (DICMAPI), University "Federico II", Piazzale Tecchio 80, 80125 Naples, Italy*

Preliminary NMR studies

For NMR studies, we reduced the total reaction volume to 1 mL of deuterated water (Table 3.1). Polymerization was performed directly in special NMR tubes designed for gel samples. Hydrogel samples were than expelled from the NMR tube and put in a solution of D_2O for the next 24h to remove all the unreacted materials. Hydrogels were dried in oven at 30°C for few hours, swelled with 1 mL of probe solution and finally put in the NMR tube for the measurement. Peak integrations demonstrate chemical structures of solution components and their related amount in the mixture. Moreover, signals attribution was confirmed by ^{1}H-NMR spectra of individual components, reported in Figure S1.

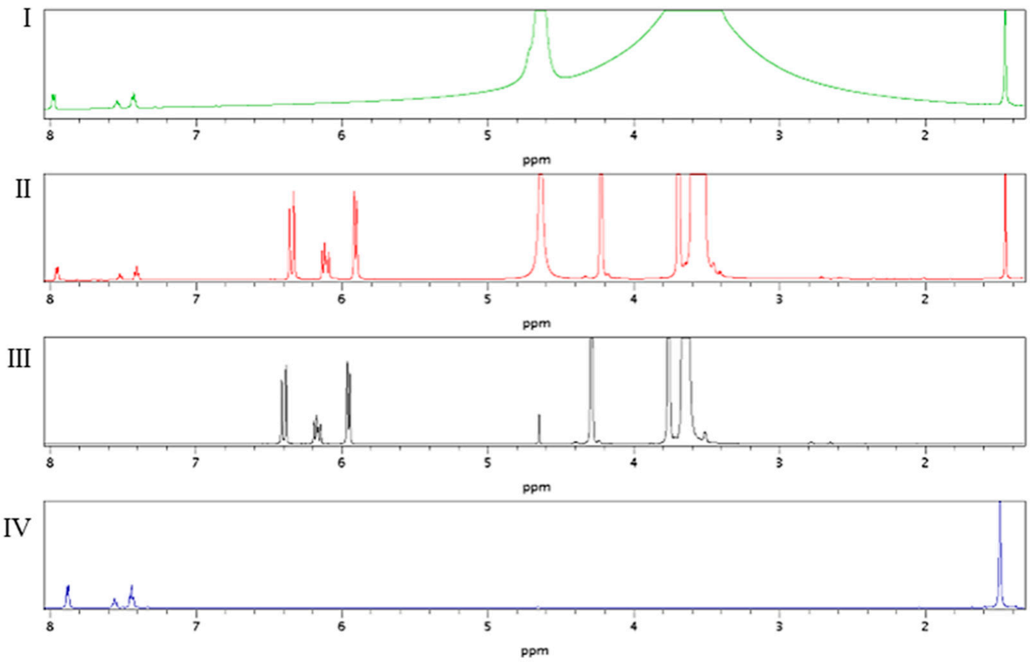

Figure S-1: ^{1}H-NMR spectra of individual components darocur (IV) and PEGDA (III) and mixture pre (II) and post- polymerization (I).

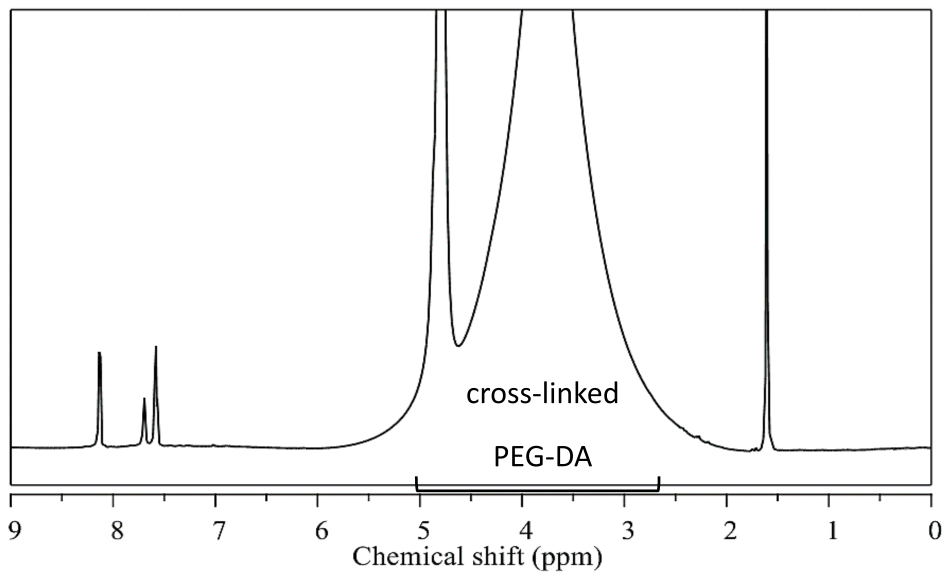

Figure S2: ^{1}H-NMR spectrum of PEGDA/darocur mixtures after polymerization.

Molecular parameters of the three-dimensional network

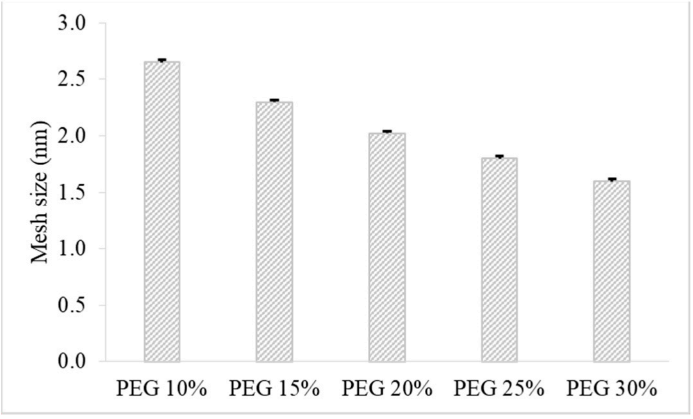

Figure S3: Mesh size values for different bulk-PEGDA concentrations

PFG-NMR

Interpolation curves fitting NMR- DOSY for water diffusion in PEGDA 10-15-20% (w/v), respectively.

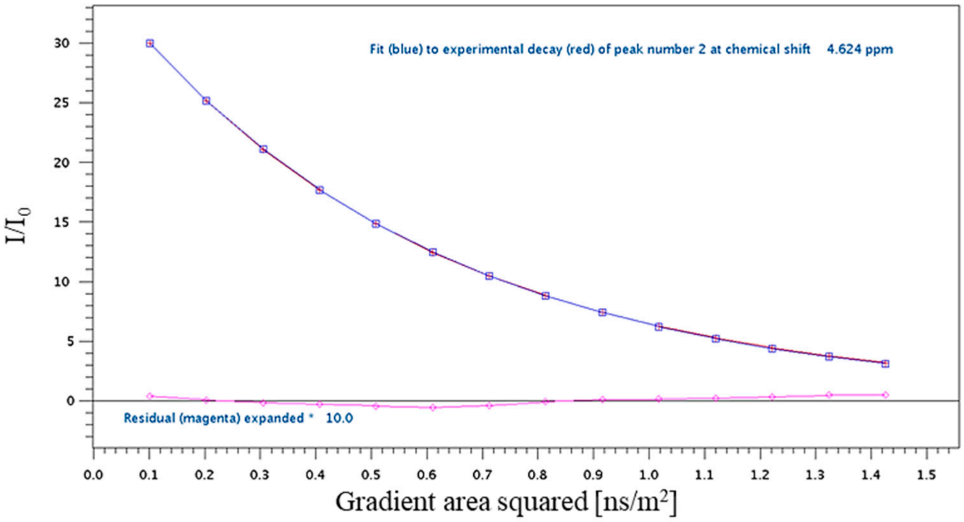

Figure S4: NMR- DOSY for water diffusion in PEGDA 10%

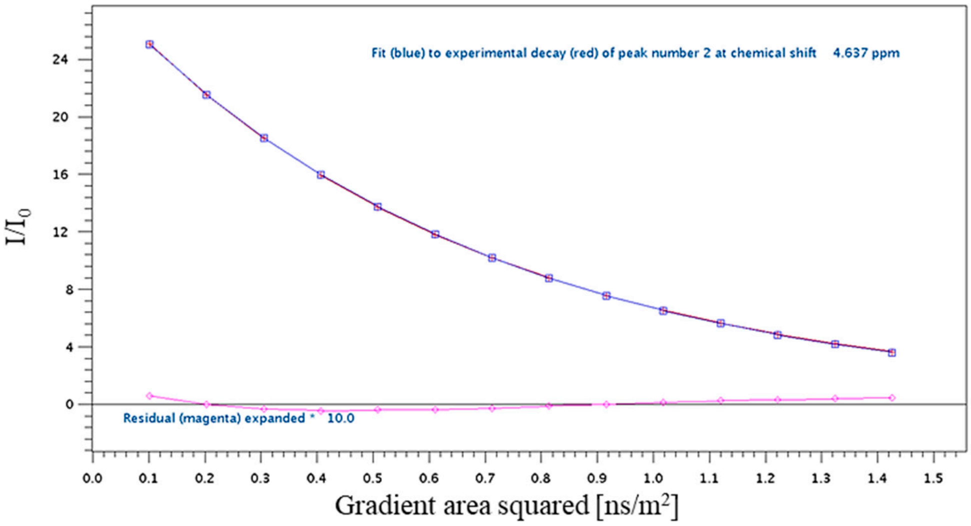

Figure S5: NMR- DOSY for water diffusion in PEGDA 15%

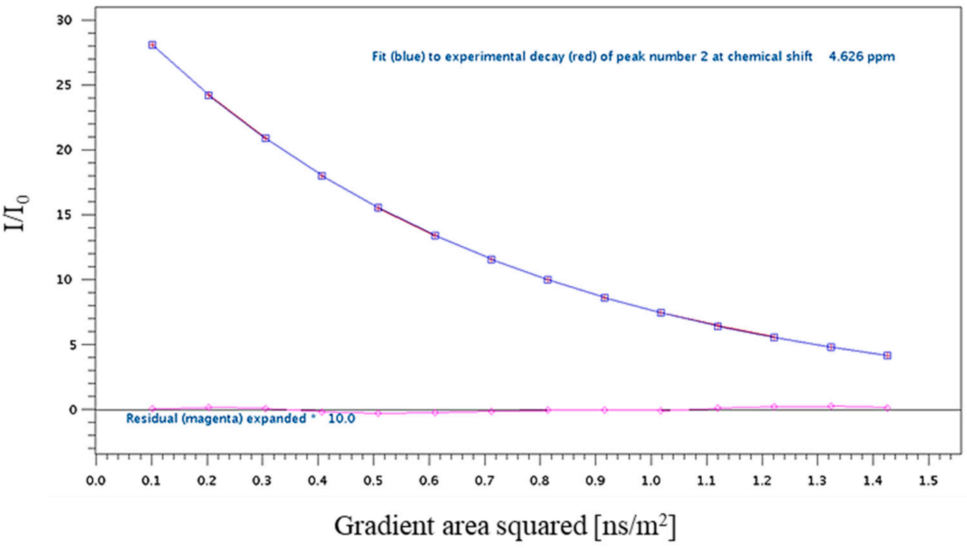

Figure S6: NMR- DOSY for water diffusion in PEGDA 20%

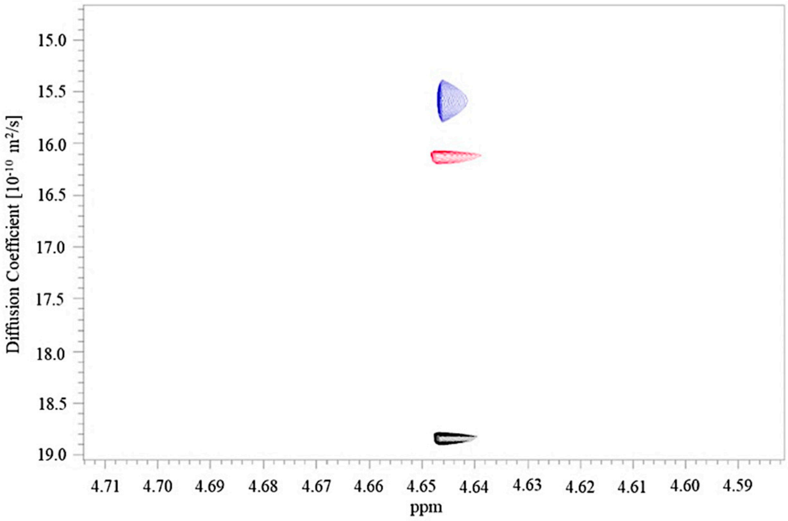

Figure S7: 2D DOSY of water (4.645 ppm) in hydrogels bulk with different PEGDA concentrations (20% in blue, 15% in red and 10% in black).

Probe optimization

For our studies, we used three DNA-sequences as reported in fluorescent T-DNA that was covalently bounded with polymer network; complementary sequence (C) for the hybridization studies and non-complementary sequence (N) as control.

Table S1 Sequence and thermodynamic parameters of the DNA probes used in this study.

probe	sequence (5'-3')	length (nt)
**Fluorescent T-DNA (F-DNA-Tail)**	TG AAA TCG GTT A	12
**Complementary sequence (C)**	T AAC CGA TTT CG ATG GTG CTA	21
**Non-complementary sequence (N)**	GAG CUA CAG UGC UUC AUC UCA	21

In order to check the quenching efficiency of fluorescent tail with quencher strand, we firstly studied this system in 10 mM PBS solution. We tested different tail concentrations (5 µM - 0.1 µM) and BHQ-strand was added 1:1 respect to the tail. Results showed a quenching percentage from ~93% to 88%. (Figure S7).

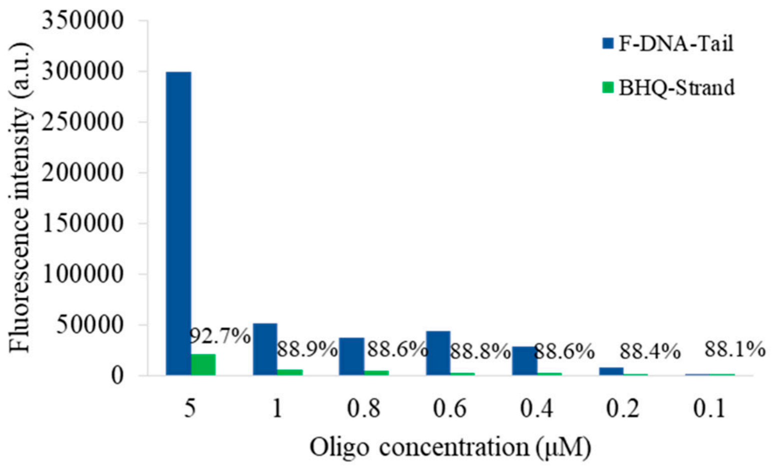

Figure S8: Quenching percentage in solution for different oligonucleotide concentrations.

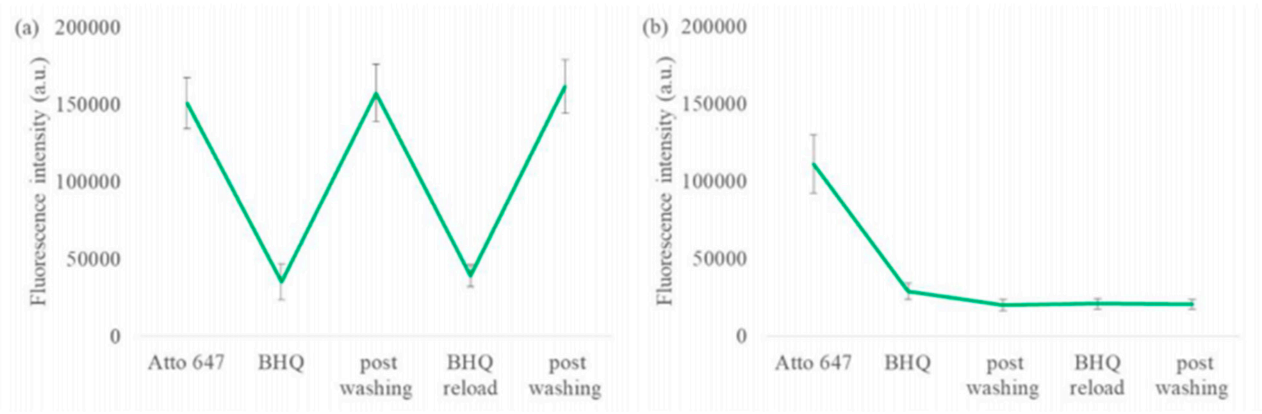

Figure S9: Fluorescence intensity of ATTO-BHQ in PEGDA 20% (a) and 10% (b).
